# The predictive ability of emotional creativity in motivation for adaptive innovation among university professors under COVID-19 epidemic: An international study

**DOI:** 10.3389/fpsyg.2022.997213

**Published:** 2022-11-03

**Authors:** Inna Čábelková, Marek Dvořák, Luboš Smutka, Wadim Strielkowski, Vyacheslav Volchik

**Affiliations:** ^1^Human Behavior Research Unit, Faculty of Economics and Management, Czech University of Life Sciences Prague (CZU), Prague, Czechia; ^2^Department of Economic Theory, Faculty of Economics, Southern Federal University, Rostov-on-Don, Russia

**Keywords:** emotional creativity, motivation, adaptive innovation, university professors, COVID-19, epidemic

## Abstract

Emotional creativity (EC) refers to cognitive abilities and personality traits related to the originality of emotional experience and expression. Previous studies have found that the COVID-19 epidemic and the restrictions imposed increased the levels of negative emotions, which obstructed adaptation. This research suggests that EC predicts the motivation for innovative adaptive behavior under the restrictions of COVID-19. In the case study of university professors, we show that EC predicts the motivation to creatively capitalize on the imposed online teaching in looking for innovative research and personal development. Methodologically, we rely on the Emotional Creativity Inventory (ECI) administered to a sample of 463 university professors (41.5% men, aged 22–100. M ± SD = 45.53 ± 11.46, median 44) from the Czech Republic (*N* = 137), Slovak Republic (*N* = 61), and Russia (*N* = 265). The indicators for motivation for innovative performance included motivations to use distant methods of scientific research, to look for partners for conducting scientific research in other cities or abroad, to conduct interdisciplinary research, starting distance learning to enhance qualifications, and the perception that due to online teaching, there is more time for personal development. We employ a set of ordinal regression analyses controlling for age, gender, position (lecturer, researcher, and manager), type of science (formal, natural, social, and applied), and country. The results suggest that Emotional Creativity and its three components predict the motivation of university professors to creatively capitalize on the imposed online teaching in looking for innovative research and personal development under the conditions of COVID-19. Furthermore, our results confirmed the gender and age differences in EC. The differences in EC according to position (lecturer, researcher, and manager) and type of science were not statistically significant. These results compel us to be aware of the importance of the emotional side of creativity to optimize stress-related behavior under the conditions of limited abilities to continue as usual. More space devoted to the manifestation of all the aspects of emotional creativity would improve adaptation to challenging circumstances and even allow one to capitalize on new opportunities. Moreover, we suggest that if personal intrinsic Emotional Creativity is high, the crises, such as the COVID-19 epidemic, may improve adaptation and trigger creative outcomes.

## Introduction

Creativity has long been a field of interest for researchers in various branches of art, sciences, and engineering (Gu et al., 2018). Generally, we distinguish cognitive and emotional processes in creative behavior (Sahin et al., 2016; He et al., 2018). Emotions are considered the biggest drivers of creative behavior in all creative endeavors, sometimes unconsciously (Gu et al., 2018). Creative adaptation is most needed in times of crisis, such as the COVID-19 epidemic caused by a novel coronavirus that emerged at the end of 2019.

The COVID-19 epidemic induced the need for novel, creative adaptation under the condition of increased stress, fear, and anxiety, and limited possibilities of action, the latter restricted by government regulations. Paradoxically, in some cases, negative moods (such as fear, anxiety, and stress) and restrictions promote creative behavior (Du et al., 2021; Mercier et al., 2021). In other cases, inherent prerequisites for creativity induced innovative adaptive outcomes (Kapoor and Kaufman, 2020). This research studies the relationship between EC and the motivation of university professors to creatively capitalize on the imposed online teaching in looking for innovative research and personal development under the conditions of COVID-19.

## Motivation for adaptive innovation

Two main types of motivation for innovation are widely studied in the literature—intrinsic and extrinsic (Fischer et al., 2019). While intrinsic motivation is generally considered beneficial, the effect of extrinsic motivation is rather controversial (Amabile et al., 1995; Anderson et al., 2014). Adaptive innovation presents the third type of motivation to innovate—the type given by the outer circumstances that make the previous behavior dysfunctional or impossible.

Kirton’s (1976) adaptive-innovative theory divides the cognitive styles of agents into adaptive (aiming to do things better) and innovative (aiming to do things differently). The preferred style is not supposed to change over life, though the opposite style may serve as a coping strategy (Buttner and Gryskiewicz, 1993; Stum, 2009). Both of these two types of behavior are necessary for adaptive innovation, though the proportions may differ.

This paper studies the effects of Emotional Creativity on the adaptive innovative behavior of university professors under the conditions of the COVID-19 epidemic. In the following chapters, we describe the problems the Higher Educational Institutions (HEIs) had to face, the effects of COVID-19 on emotional life, and the concept of Emotional Creativity. The research conducted in the following chapters studies the effects of Emotional Creativity on adaptive-innovative behavior.

## Higher educational institutions during the COVID-19 epidemic

The COVID-19 epidemic presented numerous challenges to Higher Educational Institutions (HEIs). Across the globe, lockdowns moved most of the educative and research activities online. Closed libraries, canceled (or online) conferences, and other meetings disrupted social contacts and atomized the research communities. Remote teaching brought more psychological stress to university communities (Besser et al., 2020) and caused the feeling of being overburden with new responsibilities (Zeeshan et al., 2020), in some cases eventually leading to burnout (Daumiller et al., 2021). Social distancing, enacted as a response to the COVID-19 epidemic, affected research funding by shifting the interest of researchers to the topics relevant to the COVID-19 epidemic and not requiring in-house contact with the research subjects (Gentili and Cristea, 2020; Sohrabi et al., 2021).

Job limitations enacted during the COVID-19 epidemic affected branches of science differently. Similarly, the effect of COVID restrictions on academic positions (researcher, lecturer, or manager) differed. Puranik (2021) reports that in some cases, almost 40–50% of researchers’ time was devoted to crisis management (clinical trials, employee health, sanitizing, and ensuring social distancing) rather than the research work. The COVID-19 epidemic influenced the types of research and research topics. More priorities were put on virology, epidemiology, and infectious diseases (Haleem et al., 2020; Zhang and Shaw, 2020). Apart from medical research and public health, most other research topics and procedures were significantly affected. Research contingent upon direct contact with the trial subjects suffered the most from the lockdowns as most of the research institutions were closed for at least a part of COVID-19 times. Similarly, face-to-face experiments were limited or moved to an online mode. Technical research based on laboratory measurements had to be postponed, as many of the labs were closed. Purely theoretical fundamental studies were affected, too, as they are often conducted in research groups, which were effectively moved online.

The COVID-19 epidemic also presented some opportunities. Facing impenetrable obstacles to traditional teaching and research, academics looked for new ways of research, including new areas, more use of online data collection, remote research, and wider digital dissemination of research results (Gentili and Cristea, 2020; Mseleku, 2020; Omary et al., 2020). While many viewed lockdowns as a disaster, others perceived a shift to remote teaching and research as a positive challenge that enhances their competence development (Daumiller et al., 2021). Yet others saw lockdowns as an opportunity to increase international cooperation in research (Sohrabi et al., 2021).

The opportunities for universities presented by the COVID-19 epidemic manifested themselves in four ways. First, the academics got more access to hardware/software necessary for online teaching. The universities, facing the need for online education, were buying immense amounts of relevant technologies. Second, paradoxically, some academics realized they had more time for research and creative activities after the initial overburden. Third, the university academics were deprived of the usual interactions with their colleagues and were exposed to social isolation, which might give them even more time for creative research. Finally, online teaching provided academics with more online interactions that might have motivated them to innovate in research too. In this paper, we hypothesize that Emotional Creativity might have served as a mediator in some of these processes.

## Emotions and creativity under COVID-19 restrictions

On the emotional side, the COVID-19 epidemic presented innumerable challenges. The fear of infection, the (temporary) lack of surgical masks and other equipment, limited places in the hospitals, fear of unemployment, and the enacted restrictions increased the level of stress and anxiety (Choi et al., 2020; Mann et al., 2020; Özdin and Bayrak Özdin, 2020; Wang et al., 2020). Generally, people had to struggle with fear of the unknown, social isolation, hypochondriasis, disgust, information-driven fears, and anxieties, which promoted a wide range of negative emotions (Coelho et al., 2020).

The effect of negative emotions on creativity is not clear (Davis, 2009). Generally, the effect of mood on creativity is affected by valence and arousal (He and Wong, 2022). Numerous empirical studies emphasize the supportive effects of positive moods on creative activities and ideations (Acar et al., 2021; Najafali Ghandehari et al., 2022), while negative emotions generally hinder creativity. However, some studies reported some types of negative moods could promote creativity (Akinola and Mendes, 2008; Acar et al., 2021; Du et al., 2021).

Arousal of mood is reported to be the distinguishing factor affecting the effect of negative mood on creativity (De Dreu et al., 2011; Shen et al., 2019; He and Wong, 2022). Researchers divided the types of mood into high arousal (excited: e.g., angry, fearful, and happy) and low arousal (relaxed: e.g., sad, depressed, and calm; see De Dreu et al., 2008). Some researchers believe that high arousal mood can promote creativity independently of valence (Tsai et al., 2013). Preventive-focused moods (fear and anxiety) are shown to promote creativity under some conditions too (De Dreu et al., 2008; Baas et al., 2011).

Overall, the negative mood, which appeared during the outbreak of COVID-19, was shown to be associated with cognitive creativity and emotional creativity (Du et al., 2021). The total psychological impact of the COVID-19 epidemic, being mediated *via* self-focused attention associated with the negative mood, was shown to be positively correlated to emotional creativity but not with cognitive one (ibid.).

## Emotional creativity

Emotional creativity represents one of three main areas of general creativity, together with non-verbal and verbal creativity and creativity in problem-solving (Ma, 2009). EC reflects a divergence from the ordinary emotional experience and captures originality and appropriateness in new emotional experiences (Ivcevic et al., 2007). The concept of emotional creativity originates from the social construction theory of emotion, where emotion is viewed as a transitory social role (Averill, 1980; Averill et al., 1991). Most daily emotional activities present response patterns predefined by society (Jianzhong, 2003). The critical feature of EC is a divergence from the ordinary predefined emotional experience (Ivcevic et al., 2007; Trnka et al., 2019) and to experience and express (new) emotions (Averill, 1999, 2000, 2009).

Emotional creativity includes three components: Preparedness, Novelty, and a combination of Effectiveness and Authenticity (Averill, 1999, 2000, 2009; Kuska et al., 2020). Preparedness captures specific sensitivity to emotions, and the willingness to think about, understand and explore one’s emotional reactions. The Novelty component encapsulates the ability to produce emotional responses that are novel and unique compared to socially determined responses and emotions typical to similar situations before. Effectiveness implies that new emotional reactions help solve short-term emotional problems and are beneficial for both individuals and society in the long run. The authenticity component indicates the disconnection of one’s emotional reactions from social expectations making the emotions result from self-expression rather than social expectations.

As EC involves the cognitive abilities that enable cognition to diverge from common and generate novel emotional reactions, emotionally creative abilities have been found closely related to cognitive abilities supporting innovative performance, including all the three stages of new production—idea generation, promotion, and realization (Wang et al., 2015). EC is also a significant predictor of intrinsic motivation and engagement (ibid). EC was shown to relate to self-efficacy and academic motivation (Zareie, 2014), educational adjustment, and communicative competencies (Zarenezhad et al., 2013).

Emotional creativity could help individuals cope with unfavorable circumstances by providing response flexibility in stressful situations (Frolova and Novoselova, 2015; Thomson and Jaque, 2019) and is positively correlated to mental health (Delavarpour and Lattifian, 2012). Fuchs et al. (2007) showed that EC supports behavioral self-regulation and is related to a non-confrontational, democratic, and independent personality (Ma, 2009; Tarabakina et al., 2015). In the workplace, employee creativity is considered to be a key driver of innovation and organizational success (Egan, 2005; Zhou and Hoever, 2014).

During the COVID-19 epidemic, EC improved mental health (Zhai et al., 2021) and was shown to be correlated to self-directed learning and achievement motivation (Moaser and Zarei, 2020). In this study, we hypothesize that Emotional Creativity and its components of Preparedness, Novelty, and Effectiveness/Authenticity predict the motivation of university professors to look for innovative research and personal development under the government-imposed restrictions of online teaching during the COVID-19 epidemic. Namely, online teaching itself might have motivated the professors to innovate in research.

## The study

The epidemic itself and the restrictions it brought affected the research topics and the research methods. First, the COVID-19 crisis brought the importance of interdisciplinary scholarship to the forefront (Budhwar and Cumming, 2020). The epidemic showed that originally health-related concerns initiated extensive societal changes, including political, economic, psychological, etc. The necessity for the cooperation of all the stakeholders, namely research institutions, government institutions, and business communities, substantiated the need to develop partnerships and deliver impactful research suitable for both pandemic and post-pandemic times (Beech and Anseel, 2020).

In this study, we hypothesize that Emotional Creativity and its components of Preparedness, Novelty, and Effectiveness/Authenticity positively predict the motivation of academic staff to conduct interdisciplinary research in COVID-19 as a response to online teaching (hypotheses H1.1-1.4).

The COVID-19 epidemic has further increased the importance of the international perspective as it visualized the interconnectedness of the world and the necessity of coordinated policies (Budhwar and Cumming, 2020). The need for cross-cultural validity of research methodologies, data collections, and results is more important in research (ibid). However, the isolation of some countries during COVID-19 times and the general tendency of de-globalization apparent nowadays in some supply chains bring severe doubts about the sustainability of global value chains (Verbeke, 2020). The international perspective is challenging if not impossible to achieve unless the research team is constructed of researchers based in different countries. In this study, we hypothesize that Emotional Creativity and its components of Preparedness, Novelty, and Effectiveness/Authenticity positively predict the motivation of academic staff to look for partners for conducting scientific research in other cities or abroad as a response to online teaching (H2.1.–2.4.).

The regulations related to the COVID-19 epidemic effectively limited the traditional ways of conducting research and collecting data through face-to-face methods. Many researchers had to rely on distance research methods, including secondary data analysis, online data collection, or paying firms to collect the data (Budhwar and Cumming, 2020; Lourenco and Tasimi, 2020). In this paper, we hypothesize that Emotional Creativity and its components of Preparedness, Novelty, Effectiveness/Authenticity increase the motivation of academic staff to use distant methods of scientific research as a response to online teaching (H3.1.–3.4).

The last two sets of hypotheses study the idea that online teaching brought more time for personal development and qualification enhancement (Daumiller et al., 2021). Most of the early research on online education emphasized the time necessary for designing, implementing, and conducting online courses (Fein and Logan, 2003; Humphries, 2010; Capra, 2011). Sometimes, it takes twice as long to prepare and teach classes online rather than face-to-face, thus instructors spend more time per student (Cavanaugh, 2005). In an experiment reported by Cavanaugh (2005), an economics class taught online required 155 h to prepare and teach compared with 62 h of a face-to-face course. Moreover, the time differences did not vary with the size of the class. Despite the obvious time-consuming need to move courses online, the closures of the universities, in some cases, provided more time for research. The absence of travel to and from the job left the academics more time that could be used for personal or professional development. The necessity of new computer-related skills and the increased supply of related qualification enhancement programs could make university staff work on their qualification. In this paper, we hypothesize that Emotional Creativity and its components of Preparedness, Novelty, and Effectiveness/Authenticity positively predict the motivation of academic staff to start distance learning to enhance qualification (H4.1.–4.4.). In addition, Emotional Creativity and its components of Preparedness, Novelty, and Effectiveness/Authenticity predict the belief that due to online teaching, there is more time for personal development (H5.1.–5.4.).

## Materials and methods

### Participants and procedure

Of total of 463 university professors from the Czech Republic (137 respondents), Slovak Republic (61 respondents), and Russian Federation (265 respondents), 41.5% were men, aged 22–100 years (M ± SD = 45,53 ± 11,46, median 44) completed a questionnaire voluntarily and anonymously. Three hundred sixty-six respondents (79%) had a Ph.D. (CSc.) degree and higher (Doc., full professor). The positions best describing their work duties at the universities included lecturers (338 respondents, 73%), researchers (66 respondents, 14%), and managers (59 respondents, 13%). Most respondents (329 respondents, 71,1%) worked in social sciences. Formal sciences such as logic, mathematics, and other sciences that use *a priori* rather than empirical methodology were represented by 50 respondents (10.8% of the total sample). Natural sciences, including research of natural phenomena (including cosmological, geological, physical, chemical, and biological factors), were represented by 23 respondents (5%). Applied sciences, meaning the use of scientific knowledge in a physical environment, e.g., validating theoretical models of formal science for solving a practical problem, were represented by 61 respondents (13.2%).

The method of sampling included elements of the snowball technique and opportunity sampling. The participants were recruited both personally and *via* e-mail. The research was conducted between September 2020 and January 2021.

All participants were informed that the data they are providing are confidential, will be used for research purposes only, and will not be transferred to third parties. All participants provided informed consent to participate in the study. The institutional ethics committee approved the research design.

### Materials

#### Emotional creativity

The Emotional Creativity Inventory self-report questionnaire (ECI; Averill, 1999) was employed to measure Emotional Creativity. It consists of 30 items rated on a 5-point scale ranging from 1 (strongly agree) to 5 (strongly disagree). ECI scores were computed by a simple sum of the encoded answers. Two questions were recorded to achieve higher values representing higher Emotional Creativity. The ECI scores ranged from 48 to 145 and averaged at M ± SD: 98.60 ± 15.74. The internal reliability index (Cronbach’s α) reached 0.88, which exhibits excellent internal reliability.

The ECI contains three components that reflect different aspects of Emotional Creativity. The preparedness component (range: 9–35, M ± SD: 26.28 ± 5.07, Cronbach’s α: 0.80) includes seven items like “I think about and try to understand my emotional reactions.” The novelty component (range 14–68, M ± SD: 42.27 ± 9.00, Cronbach’s α: 0.84) comprises 14 items, such as “My emotional reactions are different and unique.” The effectiveness/authenticity component (range 13–45, M ± SD: 30.05 ± 5.14, Cronbach’s α: 0.72) embraced nine items like “I respond well in situations that call for new or unusual emotional responses” or “My outward emotional reactions accurately reflect my inner feelings.” The components of Preparedness and Novelty exhibit very good reliability (Cronbach’s α of 0.86 and 0.82, respectively). The effectiveness/authenticity component exhibit acceptable reliability with Cronbach’s α of 0.72. As all the components yielded good internal reliability, they were used separately in the later analysis.

We found that EC and its components decrease with age except for the component of effectiveness/authenticity [Tables 1, 2, similar to Trnka et al. (2020)]. Women exhibited more EC as compared to men, except for the sub-scale of Novelty (Tables 1, 2).

**TABLE 1:** Pearson correlations of emotional creativity with age.

		ECI	ECIn	ECIp	ECIae
Age	Pearson correlation	−0.119*	−0.105*	−0.101*	−0.083
	Sig. (2-tailed)	0.010	0.024	0.030	0.074
	N	463	463	463	463

ECI, emotional creativity; ECIn, the ECI component of Novelty; ECIp, the ECI component or preparedness; ECIae, the ECI component of effectiveness/authenticity of emotional creativity.

***Significant at the 0.001 level (2-tailed).

**Significant at the 0.01 level (2-tailed).

*Significant at the 0.05 level (2-tailed).

**TABLE 2:** Gender differences in emotional creativity.

Gender		ECI	ECIn	ECIp	ECIae
Men	Mean	95.396	41.724	24.844	28.828
	Std. deviation	16.197	9.010	5.039	5.153
Women	Mean	100.878	42.657	27.292	30.930
	Std. deviation	15.035	8.989	4.847	4.959
	Mean difference	−5.482***	–0.933	−2.448***	−2.102***
ANOVA sig.		0.000	0.272	0.000	0.000

ECI, emotional creativity; ECIn, the ECI component of novelty; ECIp, the component or preparedness; ECIae, the component of effectiveness/authenticity of emotional creativity.

***Significant at the 0.001 level (2-tailed).

**Significant at the 0.01 level (2-tailed).

*Significant at the 0.05 level (2-tailed). *N* men = 192; *N* women = 271. Results of one-factor ANOVAs.

Surprisingly, the position (lecturer and researcher manager) was not related to EC or its components.

#### Motivation for adaptive innovation: Research and personal development

Adaptive innovation implies innovative behavior aimed to adapt to new conditions. Adaptive innovation implies that things are happening differently from what is expected, but could be beneficial (Ramalingam and Prabhu, 2020). Generally, the indicators of adaptive innovation are highly dependent on the type of organization and business processes within the organization (Cooke, 2013). In the case of Higher Educational Institutions (HEIs), the biggest change to adapt was represented by the switch to online teaching. This change produced a number of motivations in the field of research and personal development. The list of motivations studied in this paper is presented below.

Table 3 presents five indicators of motivation for innovative research and personal development. Participants were asked to indicate, on a scale of 1–5, how much they agree with the following statements:

**TABLE 3:** Indicators of motivation for research and personal development due to the COVID-19 epidemic.

1 - Strongly disagree	2 - Partially disagree	3- Hard to say	4 - Partially agree	5 - Strongly agree	Total
**1. The online teaching motivated me to use distant methods of scientific research**					
20.50%	19.20%	24.60%	23.10%	12.50%	100.00%
**2. The online teaching motivated me to look for partners for conducting scientific research in other cities or abroad.**					
32.20%	24.40%	29.20%	9.90%	4.30%	100.00%
**3. The online teaching motivated me to conduct interdisciplinary research**					
33.90%	23.80%	28.10%	9.90%	4.30%	100.00%
**4. Due to online teaching, there is more time for personal development**					
39.70%	23.80%	16.40%	13.40%	6.70%	100.00%
**5. The online teaching motivated me to start distance learning to enhance my qualification**					
25.10%	17.30%	25.70%	17.10%	14.90%	100.00%

The crosstabulations of the indicators of motivation for research and personal development vs. country are presented in Supplementary Appendix Tables 1–7. The descriptive statistics and bivariate correlations for motivations and Emotional Creativity and its sub-scales are presented in Supplementary Appendix Table 6.

(1) Online teaching motivated me to use distant methods of scientific research.
(2) Online teaching motivated me to look for partners for conducting scientific research in other cities or abroad.
(3) Online teaching motivated me to conduct interdisciplinary research.
(4) Due to online teaching, there is more time for personal development.
(5) Online teaching motivated me to start distance learning to enhance my qualification.

The crosstabulations of the indicators of motivation for research and personal development vs. country are presented in Supplementary Appendix Tables 1–7. The descriptive statistics and bivariate correlations for motivations and Emotional Creativity and its sub-scales are presented in Supplementary Appendix Tables 6, 7.

Overall, the effect of COVID-imposed online teaching on motivation to innovate in research proved to be relatively small. Only 14% of the respondents agreed (strongly or partially) that online teaching motivated them to look for partners and conduct interdisciplinary research. Thirty-five percent reported that online teaching motivated them to use distant methods of research. Conversely, the majority of the respondents did not find online teaching motivational—39–63% of respondents did not agree with the statements above (Table 3).

#### Control variables

We control for age (respondents aged 22–100; M ± SD = 45.53 ± 11.46; median 44), gender (41.5% men), and the country of residence (137 respondents from the Czech Republic, 61 from the Slovak Republic, and 265 from Russia).

As the COVID-19-related limitations may have specific effects on branches of sciences and employment positions (some sciences, for example, do not do research experiments that were banned), we control for these two variables.

Following Cohen (2021), the branches of science are defined as follows:

Formal sciences: the study of logic and mathematics, which use *a priori* rather than empirical methodology (10.8% of respondents).

Natural sciences: the study of natural phenomena (including cosmological, geological, physical, chemical, and biological factors of the universe, 5.0% of the respondents). Natural science can be divided into two main branches: physical sciences and life sciences (or biological sciences).

Social Sciences: the study of human behavior and society (71.1% of the respondents).

Applied Sciences: application of scientific knowledge transferred to the physical environment. Examples include testing a theoretical model using formal science or solving a practical problem using natural science” (13.2% of the respondents).

The employment positions were conceptualized as primarily teaching, research, or management. While the exact academic titles vary according to the country of location, these three activities are universal. Though in many cases, academics engage in all three activities, the proportion of time the academics devote to them may significantly differ. Thus, the relevant question was worded as follows:

“Please indicate the position that best describes your employment.

Lecturer: usually give lectures for students (several lectures per week, several days and a week, 73.0% of the respondents).

Researcher: writing and publishing many scientific articles and monographs (14.3% of the respondents).

Manager: directors of research centers, deans/heads of structural units, heads of the departments (12.7% of the respondents).

### Method

We rely on a set of ordinal regression analyses (formula 1)


Innovation=i

logit(aE0motionalCreativity+jaA1ge+aG2ender

(1) +aC3ountry+aS4cience+aP5osition+e)

Where

*Innovation*_*i*_—indicators of motivation for innovation presented in Table 1 subsequently: motivation to use distant methods of scientific research (*Innovation*_1_), to look for partners for conducting scientific research in other cities or abroad (*Innovation*_2_), to conduct interdisciplinary research (*Innovation*_3_), to start distance learning to enhance qualification (*Inovation*_4_), the perception that due to online teaching there is more time for personal development (*Innovation*_5_).

*Emotional Creativity_*i*_*—ECI (*Emotional Creativity_1_*), and its subscales of Novelty (*Emotional Creativity_2_*), Preparedness (*Emotional Creativity_3_*), and Effectiveness/Authenticity (*Emotional Creativity_4_*), subsequently

*Age*—age of the respondent.

*Gender*—gender of the respondent.

*Country*—country of the respondent.

*Science*—the type of science (formal, natural, social, and applied).

*Position*—the position at the university which best describes the job: lecturer, researcher, and manager.

## Results

**FIGURE 1:**
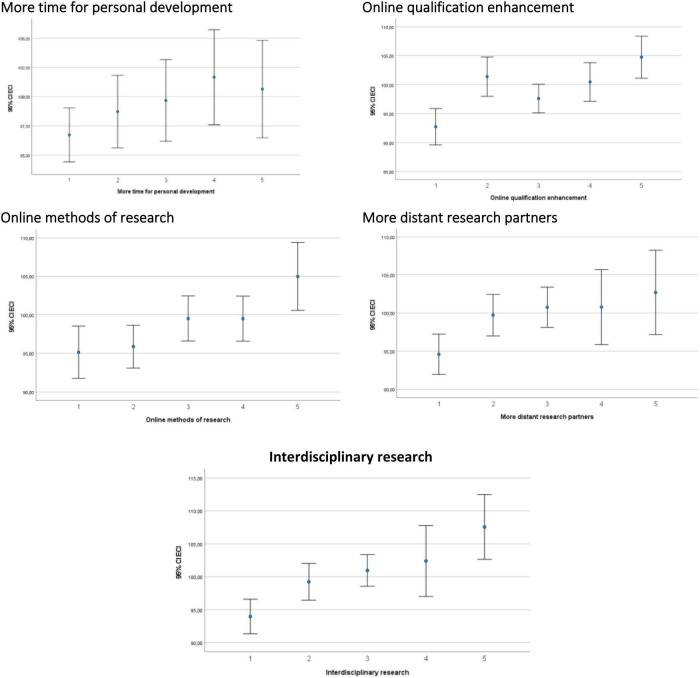
Emotional creativity inventory (ECI) as related to motivations for innovative behavior. Means and 95% confidence intervals. The scale of indicators of motivation for innovation ranges from 1—strongly disagree to 5—strongly agree.

All five indicators of motivation for innovative behavior seem to be positively related to Emotional Creativity according to the first visual analysis of means and confidence intervals presented in Figure 1. The correlation analysis presented in Supplementary Appendix Table 6 confirms the positive association. More rigorous analysis controlling for other variables is presented in Tables 4–7.

**TABLE 4:** Emotional creativity (ECI) predicts motivation for innovative research as a response to online teaching during the COVID-19 epidemic.

	Qualification enhancement		Online research		Research partner		Interdisciplinary research		Personal development	
	Estimate	Sig.	Estimate	Sig.	Estimate	Sig.	Estimate	Sig.	Estimate	Sig.
Threshold 1	1.276	0.097	−0.184	0.809	0.810	0.297	1.164	0.138	1.510	0.054
Threshold 2	2.097	0.007	0.794	0.297	1.877	0.016	2.212	0.005	2.530	0.001
Threshold 3	3.200	0.000	1.852	0.016	3.476	0.000	3.787	0.000	3.397	0.000
Threshold 4	4.226	0.000	3.248	0.000	4.795	0.000	5.134	0.000	4.688	0.000
**ECI**	**0**.024***	**0**.**000**	**0**.021***	**0**.**000**	**0**.022***	**0**.**000**	**0**.031***	**0**.**000**	**0**.017**	**0**.**003**
Age	−0.006	0.426	−0.016*	0.034	−0.005	0.526	−0.013	0.094	−0.012	0.119
Gender (men)	−0.046	0.799	−0.200	0.265	0.104	0.568	0.118	0.521	0.352	0.055
Country										
Czech Republic	−0.186	0.368	0.240	0.244	0.186	0.376	0.157	0.458	0.677**	0.001
Slovak Republic	−0.241	0.355	0.071	0.784	0.695**	0.009	0.418	0.115	0.228	0.395
Sciences										
Formal sciences	0.386	0.267	0.445	0.197	0.042	0.903	0.309	0.379	0.272	0.437
Natural sciences	0.758	0.086	−0.034	0.938	−0.262	0.557	−0.037	0.934	0.030	0.946
Social sciences	0.611*	0.017	0.151	0.546	−0.293	0.249	−0.181	0.482	0.082	0.750
Position										
Lecturer	−0.061	0.811	−0.375	0.140	−0.446	0.083	−0.735**	0.005	0.387	0.147
Researcher	−0.253	0.444	−0.008	0.981	−0.156	0.641	−0.621	0.065	0.655	0.056
Pseudo R-Square										
Cox and snell	0.067		0.059		0.060		0.090		0.065	
Nagelkerke	0.069		0.062		0.064		0.096		0.068	
McFadden	0.022		0.019		0.022		0.033		0.023	
Sig.		0.000		0.002		0.001		0.000		0.001
N	463		463		463		463		463	

Results of ordinal regression analyses. Reference variables: gender (women), country (Russian Federation), science (applied sciences), and position (manager). Link function: Logit.

***Significant at the 0.001 level (2-tailed).

**Significant at the 0.01 level (2-tailed).

*Significant at the 0.05 level (2-tailed).

**TABLE 5:** The novelty component of emotional creativity (ECIn) predicts motivation for innovative research as a response to online teaching during the COVID-19 epidemic.

	Qualificaion enhancement		Online research		Research partner		Interdisciplinary research		Personal development	
	Estimate	Sig.	Estimate	Sig.	Estimate	Sig.	Estimate	Sig.	Estimate	Sig.
Threshold 1	0.240	0.714	−1.038	0.113	0.013	0.984	0.148	0.825	0.767	0.254
Threshold 2	1.053	0.109	−0.066	0.920	1.075	0.107	1.187	0.077	1.781	0.008
Threshold 3	2.147	0.001	0.983	0.133	2.664	0.000	2.747	0.000	2.644	0.000
Threshold 4	3.165	0.000	2.372	0.000	3.980	0.000	4.088	0.000	3.929	0.000
**ECIn**	0.032**	**0.001**	0.030**	**0.002**	0.033**	**0.001**	0.048***	**0.000**	0.022*	**0.020**
Age	−0.007	0.346	−0.017*	0.026	−0.005	0.483	−0.013	0.083	−0.013	0.103
Gender (men)	−0.129	0.470	−0.272	0.127	0.019	0.915	0.010	0.955	0.285	0.118
Country										
Czech Republic	−0.233	0.255	0.197	0.335	0.156	0.453	0.107	0.609	0.640**	0.002
Slovak republic	−0.222	0.394	0.080	0.758	0.703**	0.008	0.429	0.106	0.242	0.366
Sciences										
Formal sciences	0.441	0.204	0.480	0.164	0.089	0.799	0.368	0.293	0.295	0.399
Natural sciences	0.800	0.069	0.011	0.980	−0.202	0.650	0.032	0.942	0.079	0.859
Social sciences	0.645*	0.011	0.183	0.464	−0.245	0.334	−0.129	0.615	0.106	0.680
Position										
Lecturer	−0.040	0.875	−0.360	0.155	−0.442	0.086	−0.709**	0.006	0.389	0.145
Researcher	−0.239	0.470	−0.011	0.973	−0.150	0.654	−0.611	0.069	0.649	0.057
Pseudo R-Square										
Cox and Snell	0.053		0.050		0.053		0.081		0.057	
Nagelkerke	0.056		0.052		0.056		0.086		0.060	
McFadden	0.017		0.016		0.019		0.030		0.020	
Sig.		0.005		0.008		0.005		0.000		0.002
N		463		463		463		463		463

Results of ordinal regression analyses. Reference variables: gender (women), country (Russian Federation), science (applied sciences), and position (manager). Link function: Logit.

***Significant at the 0.001 level (2−tailed).

**Significant at the 0.01 level (2-tailed).

*Significant at the 0.05 level (2-tailed).

**TABLE 6:** The preparedness component of emotional creativity (ECIp) predicts motivation for innovative research as a response to online teaching during the COVID-19 epidemic.

	Qualificaion enhancement		Online research		Research partner		Interdisciplinary research		Personal development	
	Estimate	Sig.	Estimate	Sig.	Estimate	Sig.	Estimate	Sig.	Estimate	Sig.
Threshold 1	0.654	0.346	−0.607	0.378	−0.119	0.865	−0.165	0.815	0.737	0.296
Threshold 2	1.471	0.035	0.373	0.587	0.932	0.183	0.857	0.224	1.747	0.014
Threshold 3	2.571	0.000	1.430	0.038	2.514	0.000	2.404	0.001	2.606	0.000
Threshold 4	3.594	0.000	2.822	0.000	3.830	0.000	3.738	0.000	3.893	0.000
**ECIp**	0.069***	**0.000**	0.065***	**0.000**	0.048**	**0.007**	0.069***	**0.000**	**0.035**	**0.050**
Age	−0.007	0.368	−0.017*	0.025	−0.006	0.449	−0.015	0.052	−0.013	0.092
Gender (men)	−0.016	0.928	−0.165	0.359	0.103	0.573	0.123	0.503	0.355	0.054
**Country**										
Czech Republic	−0.169	0.415	0.281	0.176	0.175	0.406	0.119	0.576	0.670**	0.002
Slovak Republic	−0.163	0.533	0.147	0.574	0.753**	0.005	0.485	0.067	0.281	0.294
**Science**										
Formal sciences	0.381	0.273	0.454	0.187	0.089	0.798	0.319	0.362	0.281	0.422
Natural sciences	0.765	0.083	−0.026	0.953	−0.256	0.566	−0.074	0.869	0.039	0.930
Social sciences	0.564*	0.027	0.137	0.586	−0.274	0.282	−0.194	0.450	0.084	0.746
**Position**										
Lecturer	−0.108	0.671	−0.415	0.103	−0.447	0.083	−0.741**	0.004	0.377	0.158
Researcher	−0.253	0.444	−0.007	0.984	−0.114	0.733	−0.597	0.076	0.670*	0.050
Pseudo R-Square										
Cox and Snell	0.061		0.058		0.044		0.062		0.053	
Nagelkerke	0.064		0.060		0.047		0.066		0.057	
McFadden	0.020		0.019		0.016		0.022		0.019	
Sig		0.001		0.002		0.022		0.001		0.005
N	463		463		463		463		463	

Results of ordinal regression analyses. Reference Variables: gender (women), country (Russian Federation), science (applied sciences), and position (manager). Link function: Logit.

***Significant at the 0.001 level (2-tailed).

**Significant at the 0.01 level (2-tailed).

*Significant at the 0.05 level (2-tailed).

**TABLE 7:** The Effectiveness/Authenticity component of emotional Creativity (ECIea) predicts motivation for innovative research as a response to online teaching during the COVID-19 epidemic.

	Qualification enhancement		Online research		Research partner		Interdisciplinary research		Personal development	
	Estimate	Sig.	Estimate	Sig.	Estimate	Sig.	Estimate	Sig.	Estimate	Sig.
Threshold 1	0.462	0.528	−1.033	0.157	0.274	0.712	0.127	0.865	1.595	0.034
Threshold 2	1.275	0.082	−0.068	0.926	1.332	0.073	1.156	0.121	2.619	0.001
Threshold 3	2.375	0.001	0.980	0.179	2.928	0.000	2.714	0.000	3.489	0.000
Threshold 4	3.392	0.000	2.368	0.001	4.246	0.000	4.045	0.000	4.783	0.000
**ECIea**	0.054**	**0.002**	0.042*	**0.012**	0.056**	**0.001**	0.067***	**0.000**	0.057**	**0.001**
Age	−0.008	0.311	−0.017*	0.020	−0.006	0.411	−0.014	0.069	−0.012	0.116
Gender (men)	−0.025	0.892	−0.188	0.298	0.131	0.477	0.109	0.556	0.405*	0.029
Country										
Czech Republic	−0.288	0.157	0.141	0.487	0.095	0.647	0.050	0.810	0.617**	0.003
Slovak Republic	−0.322	0.218	−0.011	0.966	0.619*	0.020	0.328	0.216	0.141	0.599
Sciences										
Formal sciences	0.383	0.270	0.453	0.188	0.053	0.879	0.272	0.437	0.237	0.499
Natural sciences	0.797	0.070	−0.002	0.997	−0.224	0.616	−0.031	0.944	0.020	0.965
Social sciences	0.623*	0.014	0.177	0.478	−0.268	0.291	−0.169	0.507	0.080	0.756
Position										
Lecturer	−0.022	0.930	−0.332	0.190	−0.392	0.128	−0.668*	0.010	0.436	0.103
Researcher	−0.228	0.490	0.002	0.994	−0.116	0.728	−0.562	0.093	0.674*	0.049
Pseudo R-Square										
Cox and Snell	0.051		0.043		0.051		0.064		0.069	
Nagelkerke	0.053		0.045		0.054		0.068		0.073	
McFadden	0.017		0.014		0.018		0.023		0.025	
Sig		0.007		0.027		0.007		0.001		0.000
N	463		463		463		463		463	

Results of ordinal regression analyses. Reference Variables: gender (women), country (Russian Federation), science (applied sciences), and position (manager). Link function: Logit.

***Significant at the 0.001 level (2-tailed).

**Significant at the 0.01 level (2-tailed).

*Significant at the 0.05 level (2-tailed).

Tables 4–7 present the results of ordinal regression analyses (formula 1). The descriptive statistics and correlations are presented in Supplementary Appendix Tables 1–7. Table 8 summarizes the results presented in Tables 4–7.

**TABLE 8:** Summary of the associations between emotional creativity and indicators of adaptive innovations presented in Tables 4–7.

	Qualification enhancement	Online research	Research partner	Interdisciplinary research	Personal development
ECI	+	+	+	+	+
ECIn	+	+	+	+	+
ECIp	+	+	+	+	
ECIae	+	+	+	+	+

Results of ordinal regression analyses (formula 1). + implies that the association is statistically significant on conventional levels and the sign of the relationship is positive. The association between ECIp and Personal development was at the edge of significance, thus it is not presented here.

The results confirmed that Emotional Creativity and all its components predicted motivation for innovative research and personal development as a response to the imposed online teaching during the COVID-19 epidemic (Tables 4–8). The results proved robust with respect to employed indicators of motivation for innovative behavior, except the component of preparedness related to more time for personal development, which was at the edge of significance.

Age negatively predicted the motivation for online research in all the regression models—older people usually have more trouble with computer-related remote technologies.

The results suggest significant gender differences in the time available for personal development as a response to online teaching—men reported finding more time for personal development (Table 7, otherwise at the edge of significance).

In the Czech Republic, academics reported time for personal development as a response to imposed online teaching. Slovaks were more motivated to look for research partners in other cities or abroad.

Academics working in Social Sciences were more inclined to start distance learning for qualification enhancement. Understandably, lecturers were less motivated to conduct interdisciplinary research as their primary duties place more attention on teaching than on research.

## Discussion

The results elucidate the role of Emotional Creativity in motivation for innovation under the extreme conditions represented by the epidemic of COVID-19. From theory, creativity should help to look for more innovative ways to overcome difficulties if it is not suppressed by stress and anxiety. However, the unprecedented negative emotions evoked by the epidemic could also enhance creativity, though usually suppressing it (Akinola and Mendes, 2008; Acar et al., 2021; Du et al., 2021)—some researchers believe that high arousal mood can promote creativity independently of valence (Tsai et al., 2013). In addition, preventive-focused moods (fear, anxiety, and frequency during the epidemic) are shown to promote creativity under some conditions (De Dreu et al., 2008; Baas et al., 2011).

Though we do not have information about the change in creativity indicators throughout the epidemic, this research suggests that the levels of EC were still sufficient to differentiate between the people with motivation to more and less innovative behavior.

From existing knowledge, EC is not easy to alter. While Emotional Intelligence can be, to a large extent, learned (Zeidner et al., 2003; Vernon et al., 2009; Nelson and Low, 2011; Keefer et al., 2018), successful attempts to change Emotional Creativity are rather rare (Mahasneh and Gazo, 2019; Yeh et al., 2019; Seyyedan et al., 2020). On the other hand, Emotional Creativity is known to be diminishing with age (Trnka et al., 2020), related to cognitive functions (Trnka et al., 2019), and differs according to gender (women have higher EC, Trnka et al., 2020). Our results suggest that it is one of the significant predictors of motivation for innovative behavior even throughout the epidemic. The significance of all three components of EC incites the discussion on how emotional creativity supports innovation.

Significant gender differences in the time available for personal development were manifested as a response to online teaching. Men reported more time for personal development (Table 7, otherwise at the edge of significance). Supposedly, women had to carry heavier burdens of taking care of households and kids who were no longer in school; thus, they used their time otherwise. Similar to the above, Pinho-Gomes et al. (2020) showed that women were very underrepresented in research authorship during the COVID-19 epidemic.

The results presented in this paper are subjected to the following limitations. First, the method of sampling included the elements of the snowball technique, opportunity sampling. Thus, the resulting sample cannot be considered representative. On the other hand, the sample presented considerable variability in socio-demographic and other parameters of the respondents, which, afterward were controlled in the analysis. The second limitation of this study is that we did not control for the emotional state of the respondents, which could be significantly altered by the COVID-19 epidemic. The effect of the emotional state as a mediating effect for the studied relationship can be proposed for future research.

## Conclusion

Creativity generally supports creative performance and innovations (Fetrati and Nielsen, 2018; Alzoubi et al., 2021). However, negative emotions and stress present over the COVID-19 epidemic might have mitigated the impact of creativity on innovation. This paper studied the effects of Emotional Creativity (EC) and its components of Preparedness, Novelty, and Effectiveness/Authenticity on motivation for innovation under limiting conditions of the COVID-19 epidemic on the motivation of innovative behavior utilizing the case study of university professors under the restrictions of the COVID-19 epidemic.

The need for creative, innovative behavior is particularly urgent under extreme and limiting life experiences when one cannot continue as usual. The COVID-19-related regulations highly limited the functioning of Higher Educational Institutions (HEIs). The academics had to look for new ways of functioning under the new conditions. Many of them experienced increasing psychological stress and overburden, eventually leading to burnout (Besser et al., 2020; [Bibr B77][Bibr B22]). Others viewed the COVID-19-induced limitation as an opportunity for self-education and innovation (Gentili and Cristea, 2020; Mseleku, 2020; Omary et al., 2020). From this point of view, the epidemic of COVID-19 served as a trigger to innovations, but only for some. Our results suggest that Emotional Creativity served as a differentiating factor distinguishing those suffering from restrictions from those viewing them as an opportunity to innovate.

Our findings contribute to the research on Emotional Creativity, literature on individual motivation and innovative behavior under limiting circumstances, and the role of crises as a trigger for innovative behavior.

## Data availability statement

The raw data supporting the conclusions of this article will be made available by the authors, without undue reservation.

## Ethics statement

The studies involving human participants were reviewed and approved by the Ethics Committee of the Czech University of Life Sciences. The patients/participants provided their written informed consent to participate in this study.

## Author contributions

IČ, WS, and LS developed the research idea together and designed the questionnaire. MD, LS, and VV collected the data for this article. IČ analyzed the data and drafted the manuscript. WS, LS, and MD provided the critical revisions. All authors contributed to the article and approved the submitted version.
