# Supplementary material for: The predictive ability of emotional creativity in motivation for adaptive innovation among university professors under COVID-19 epidemic: An international study
Source: Front Psychol. 2022 Nov 3;13:997213. doi: 10.3389/fpsyg.2022.997213 (PMC9669899; doi:10.3389/fpsyg.2022.997213)
Supplement: Supplementary file 1 [file Data_Sheet_1.docx]

# Appendix.

Table A1. Motivation to start distance learning to enhance qualification. Crosstabulations of responses in countries.

|  |  |  | 1-disagree^a^ | 2^a^ | 3^a^ | 4^a^ | 5-agree^a^ | Total |
| --- | --- | --- | --- | --- | --- | --- | --- | --- |
| country | Czech Republic | Count | 41 | 21 | 41 | 21 | 13 | 137 |
|  |  | % within country | 29,90% | 15,30% | 29,90% | 15,30% | 9,50% | 100,00% |
|  | Slovak Republic | Count | 17 | 7 | 21 | 8 | 8 | 61 |
|  |  | % within country | 27,90% | 11,50% | 34,40% | 13,10% | 13,10% | 100,00% |
|  | Russian Federation | Count | 58 | 52 | 57 | 50 | 48 | 265 |
|  |  | % within country | 21,90% | 19,60% | 21,50% | 18,90% | 18,10% | 100,00% |
| Total |  | Count | 116 | 80 | 119 | 79 | 69 | 463 |
|  |  | % within country | 25,10% | 17,30% | 25,70% | 17,10% | 14,90% | 100,00% |

^a^ Online teaching motivated me to start distance learning to enhance my qualification, 1—strongly disagree and5—strongly agree.

Table A2. Motivation to use distant methods of scientific research. Crosstabulations of responses in countries.

|  |  |  | 1-disagree^a^ | 2^a^ | 3^a^ | 4^a^ | 5-agree^a^ | Total |
| --- | --- | --- | --- | --- | --- | --- | --- | --- |
| country | Czech Republic | Count | 22 | 31 | 41 | 28 | 15 | 137 |
|  |  | % within country | 16,10% | 22,60% | 29,90% | 20,40% | 10,90% | 100,00% |
|  | Slovak Republic | Count | 8 | 15 | 19 | 16 | 3 | 61 |
|  |  | % within country | 13,10% | 24,60% | 31,10% | 26,20% | 4,90% | 100,00% |
|  | Russian Federation | Count | 65 | 43 | 54 | 63 | 40 | 265 |
|  |  | % within country | 24,50% | 16,20% | 20,40% | 23,80% | 15,10% | 100,00% |
| Total |  | Count | 95 | 89 | 114 | 107 | 58 | 463 |
|  |  | % within country | 20,50% | 19,20% | 24,60% | 23,10% | 12,50% | 100,00% |

^a^ Online teaching motivated me to use distant methods of conducting scientific research, 1—strongly disagree and5—strongly agree.

Table A3. Motivation to look for partners for conducting scientific research in other cities or abroad. Crosstabulations of responses in countries.

|  |  |  | 1-disagree^a^ | 2^a^ | 3^a^ | 4^a^ | 5-agree^a^ | Total |
| --- | --- | --- | --- | --- | --- | --- | --- | --- |
| country | Czech Republic | Count | 44 | 37 | 34 | 17 | 5 | 137 |
|  |  | % within country | 32,10% | 27,00% | 24,80% | 12,40% | 3,60% | 100,00% |
|  | Slovak Republic | Count | 10 | 13 | 29 | 9 | 0 | 61 |
|  |  | % within country | 16,40% | 21,30% | 47,50% | 14,80% | 0,00% | 100,00% |
|  | Russian Federation | Count | 95 | 63 | 72 | 20 | 15 | 265 |
|  |  | % within country | 35,80% | 23,80% | 27,20% | 7,50% | 5,70% | 100,00% |
| Total |  | Count | 149 | 113 | 135 | 46 | 20 | 463 |
|  |  | % within country | 32,20% | 24,40% | 29,20% | 9,90% | 4,30% | 100,00% |

^a^ The distance form of teaching motivated me to look for partners for conducting scientific research in other cities or abroad, 1—strongly disagree and5—strongly agree.

Table A4. Motivation to conduct interdisciplinary research. Crosstabulations of responses in countries.

|  |  |  | 1-disagree^a^ | 2^a^ | 3^a^ | 4^a^ | 5-agree^a^ | Total |
| --- | --- | --- | --- | --- | --- | --- | --- | --- |
| country | Czech Republic | Count | 48 | 32 | 36 | 16 | 5 | 137 |
|  |  | % within country | 35,00% | 23,40% | 26,30% | 11,70% | 3,60% | 100,00% |
|  | Slovak Republic | Count | 13 | 18 | 21 | 7 | 2 | 61 |
|  |  | % within country | 21,30% | 29,50% | 34,40% | 11,50% | 3,30% | 100,00% |
|  | Russian Federation | Count | 96 | 60 | 73 | 23 | 13 | 265 |
|  |  | % within country | 36,20% | 22,60% | 27,50% | 8,70% | 4,90% | 100,00% |
| Total |  | Count | 157 | 110 | 130 | 46 | 20 | 463 |
|  |  | % within country | 33,90% | 23,80% | 28,10% | 9,90% | 4,30% | 100,00% |

^a^ The distance form of teaching motivated me to conduct interdisciplinary research, 1—strongly disagree, 5—strongly agree.

Table A5. Time for personal development. Crosstabulations of responses in countries

|  |  |  | 1-disagree^a^ | 2^a^ | 3^a^ | 4^a^ | 5-agree^a^ | Total |
| --- | --- | --- | --- | --- | --- | --- | --- | --- |
| country | Czech Republic | Count | 40 | 35 | 27 | 21 | 14 | 137 |
|  |  | % within country | 29,20% | 25,50% | 19,70% | 15,30% | 10,20% | 100,00% |
|  | Slovak Republic | Count | 22 | 19 | 11 | 6 | 3 | 61 |
|  |  | % within country | 36,10% | 31,10% | 18,00% | 9,80% | 4,90% | 100,00% |
|  | Russian Federation | Count | 122 | 56 | 38 | 35 | 14 | 265 |
|  |  | % within country | 46,00% | 21,10% | 14,30% | 13,20% | 5,30% | 100,00% |
| Total |  | Count | 184 | 110 | 76 | 62 | 31 | 463 |
|  |  | % within country | 39,70% | 23,80% | 16,40% | 13,40% | 6,70% | 100,00% |

^a^ Due to online teaching, there is more time for personal development, 1—strongly disagree and 5—strongly agree.

**Table A6. Pearson correlations for indicators of motivation and ECI, including subscales.**

|  |  | (1) | (2) | (3) | (4) | (5) | (6) | (7) | (8) | (9) | (10) | (11) | (12) | (13) |
| --- | --- | --- | --- | --- | --- | --- | --- | --- | --- | --- | --- | --- | --- | --- |
| Age (1) | Corr. | 1,000 | -0,069 | -,126** | -0,010 | -0,075 | -0,087 | -,119* | -,105* | -,101* | -0,083 | ,116* | 0,049 | -0,116 |
|  | Sig. |  | 0,139 | 0,006 | 0,824 | 0,105 | 0,061 | 0,010 | 0,024 | 0,030 | 0,074 | 0,012 | 0,298 | 0,103 |
| **Motivation for** |  |  |  |  |  |  |  |  |  |  |  |  |  |  |
| Qualification enhancement (2) | Corr. | -0,069 | 1,000 | ,535** | ,367** | ,426** | ,234** | ,212** | ,164** | ,210** | ,156** | ,138** | 0,035 | -0,009 |
|  | Sig. | 0,139 |  | 0,000 | 0,000 | 0,000 | 0,000 | 0,000 | 0,000 | 0,000 | 0,001 | 0,003 | 0,447 | 0,896 |
| Online research (3) | Corr. | -,126** | ,535** | 1,000 | ,516** | ,573** | ,213** | ,181** | ,149** | ,169** | ,127** | ,096* | ##### | -0,101 |
|  | Sig. | 0,006 | 0,000 |  | 0,000 | 0,000 | 0,000 | 0,000 | 0,001 | 0,000 | 0,006 | 0,040 | 0,606 | 0,158 |
| Partners abroad (4) | Corr. | -0,010 | ,367** | ,516** | 1,000 | ,699** | ,183** | ,160** | ,139** | ,099* | ,149** | ,159** | 0,056 | -0,070 |
|  | Sig. | 0,824 | 0,000 | 0,000 |  | 0,000 | 0,000 | 0,001 | 0,003 | 0,032 | 0,001 | 0,001 | 0,232 | 0,327 |
| Interdisciplinary research (5) | Corr. | -0,075 | ,426** | ,573** | ,699** | 1,000 | ,202** | ,228** | ,212** | ,155** | ,176** | ,149** | 0,047 | -0,067 |
|  | Sig. | 0,105 | 0,000 | 0,000 | 0,000 |  | 0,000 | 0,000 | 0,000 | 0,001 | 0,000 | 0,001 | 0,316 | 0,347 |
| Personal development (more time, 6) | Corr. | -0,087 | ,234** | ,213** | ,183** | ,202** | 1,000 | ,107* | 0,087 | 0,044 | ,131** | ,134** | -,091* | -0,047 |
|  | Sig. | 0,061 | 0,000 | 0,000 | 0,000 | 0,000 |  | 0,022 | 0,062 | 0,346 | 0,005 | 0,004 | 0,050 | 0,515 |
| **Indicators of EC** |  |  |  |  |  |  |  |  |  |  |  |  |  |  |
| ECI (7) | Corr. | -,119* | ,212** | ,181** | ,160** | ,228** | ,107* | 1,000 | ,889** | ,769** | ,748** | 0,053 | 0,077 | -0,038 |
|  | Sig. | 0,010 | 0,000 | 0,000 | 0,001 | 0,000 | 0,022 |  | 0,000 | 0,000 | 0,000 | 0,256 | 0,098 | 0,592 |
| ECIn (8) | Corr. | -,105* | ,164** | ,149** | ,139** | ,212** | 0,087 | ,889** | 1,000 | ,511** | ,469** | 0,026 | 0,070 | -0,110 |
|  | Sig. | 0,024 | 0,000 | 0,001 | 0,003 | 0,000 | 0,062 | 0,000 |  | 0,000 | 0,000 | 0,577 | 0,132 | 0,124 |
| ECIp (9) | Corr. | -,101* | ,210** | ,169** | ,099* | ,155** | 0,044 | ,769** | ,511** | 1,000 | ,476** | 0,041 | 0,034 | 0,050 |
|  | Sig. | 0,030 | 0,000 | 0,000 | 0,032 | 0,001 | 0,346 | 0,000 | 0,000 |  | 0,000 | 0,383 | 0,463 | 0,488 |
| ECIae (10) | Corr. | -0,083 | ,156** | ,127** | ,149** | ,176** | ,131** | ,748** | ,469** | ,476** | 1,000 | 0,076 | 0,080 | 0,024 |
|  | Sig. | 0,074 | 0,001 | 0,006 | 0,001 | 0,000 | 0,005 | 0,000 | 0,000 | 0,000 |  | 0,101 | 0,087 | 0,737 |
| **Other factors** |  |  |  |  |  |  |  |  |  |  |  |  |  |  |
| Difficulties with technologies (11) | Corr. | ,116* | ,138** | ,096* | ,159** | ,149** | ,134** | 0,053 | 0,026 | 0,041 | 0,076 | 1,000 | ,283** | 0,095 |
|  | Sig. | 0,012 | 0,003 | 0,040 | 0,001 | 0,001 | 0,004 | 0,256 | 0,577 | 0,383 | 0,101 |  | 0,000 | 0,185 |
| Lack of equipment (12) | Corr. | 0,049 | 0,035 | -0,024 | 0,056 | 0,047 | -,091* | 0,077 | 0,070 | 0,034 | 0,080 | ,283** | 1,000 | ,165* |
|  | Sig. | 0,298 | 0,447 | 0,606 | 0,232 | 0,316 | 0,050 | 0,098 | 0,132 | 0,463 | 0,087 | 0,000 |  | 0,020 |
| Experience with online teaching (13) | Corr. | -0,116 | -0,009 | -0,101 | -0,070 | -0,067 | -0,047 | -0,038 | -0,110 | 0,050 | 0,024 | 0,095 | ,165* | 1,000 |
|  | Sig. | 0,103 | 0,896 | 0,158 | 0,327 | 0,347 | 0,515 | 0,592 | 0,124 | 0,488 | 0,737 | 0,185 | 0,020 |  |

Note: Pearson correlation. Sig. is 2-tailed. *N* = 463.

**Table A7. Descriptive statistics of indicators of motivation and ECI, including sub-scales.**

|  | Mean | Std. Deviation | N |
| --- | --- | --- | --- |
| Qualification enhancement | 2,790 | 1,380 | 463 |
| Online research | 2,880 | 1,317 | 463 |
| Partners for research | 2,300 | 1,146 | 463 |
| Interdisciplinary research | 2,270 | 1,156 | 463 |
| Personal development | 2,240 | 1,284 | 463 |
| ECI | 98,605 | 15,744 | 463 |
| ECIn | 42,270 | 9,000 | 463 |
| ECIp | 26,277 | 5,068 | 463 |
| ECIae | 30,058 | 5,140 | 463 |
